# The impact of non-genetic and genetic factors on a stable warfarin dose in Thai patients

**DOI:** 10.1007/s00228-017-2265-8

**Published:** 2017-05-26

**Authors:** Nitsupa Wattanachai, Sutthida Kaewmoongkun, Burabha Pussadhamma, Pattarapong Makarawate, Chaiyasith Wongvipaporn, Songsak Kiatchoosakun, Suda Vannaprasaht, Wichittra Tassaneeyakul

**Affiliations:** 1 Department of Pharmacology, Faculty of Medicine, Khon Kaen University, Khon Kaen, 40002 Thailand; 2 Division of Cardiology, Queen Sirikit Heart Center of the Northeast, Department of Medicine, Faculty of Medicine, Khon Kaen University, Khon Kaen, 40002 Thailand

**Keywords:** Non-genetic factors, *CYP2C9*, *CYP4F2*, *VKORC1*, *UGT1A1*, Warfarin

## Abstract

**Purpose:**

The aim of this study was to investigate the contributions of non-genetic and genetic factors on the variability of stable warfarin doses in Thai patients.

**Methods:**

A total of 250 Thai patients with stable warfarin doses were enrolled in the study. Demographics and clinical data, e.g., age, body mass index, indications for warfarin and concomitant medications, were documented. Four single nucleotide polymorphisms in the *VKORC1* − 1639G > A, CYP2C9*3, *CYP4F2* rs2108622, and *UGT1A1* rs887829 genes were detected from gDNA using TaqMan allelic discrimination assays.

**Results:**

The patients with variant genotypes of *VKORC1* − 1639G > A required significantly lower warfarin stable weekly doses (SWDs) than those with wild-type genotype (*p* < 0.001). Similarly, the patients with CYP2C9*3 variant allele required significantly lower warfarin SWDs than those with homozygous wild-type (*p* = 0.006). In contrast, there were no significant differences in the SWDs between the patients who carried variant alleles of *CYP4F2* rs2108622 and *UGT1A1* rs887829 as compared to wild-type allele carriers. Multivariate analysis, however, showed that *CYP4F2* rs2108622 TT genotype accounted for a modest part of warfarin dose variability (1.2%). In contrast, *VKORC1* − 1639G > A, CYP2C9*3, *CYP4F2* rs2108622 genotypes and non-genetic factors accounted for 51.3% of dose variability.

**Conclusions:**

*VKORC1* − 1639G > A, CYP2C9*3, and *CYP4F2* rs2108622 polymorphisms together with age, body mass index, antiplatelet drug use, amiodarone use, and current smoker status explained 51.3% of individual variability in stable warfarin doses. In contrast, the *UGT1A1* rs887829 polymorphism did not contribute to dose variability.

**Electronic supplementary material:**

The online version of this article (doi:10.1007/s00228-017-2265-8) contains supplementary material, which is available to authorized users.

## Introduction

Warfarin is a widely used oral anticoagulant for the prevention and treatment of various thromboembolic diseases including atrial fibrillation, heart valve replacement, pulmonary embolism, and deep vein thrombosis [1]. Due to its narrow therapeutic index and the large inter-individual variation of warfarin response, dosing may result in serious complications associated with bleeding or thromboembolism [2]. Several genetic and non-genetic factors, e.g., age and body mass index, have been reported to be associated with warfarin dose requirements [3]. Genetic polymorphisms are involved in both the pharmacokinetics and pharmacodynamics of warfarin and, therefore, appear to play an important role in the inter-individual variability of warfarin doses [4]. Two important genes have been identified as being responsible for warfarin treatment, including *cytochrome P450 2C9 (CYP2C9)* and the *vitamin K epoxide reductase complex subunit 1* (*VKORC1*) [5]. *CYP2C9* gene encodes the CYP2C9 enzyme, which is the main metabolizing enzyme of *S*-warfarin. The CYP2C9*2 and CYP2C9*3 variants have been shown to have decreased enzymatic activity, which partly accounts for the variance in warfarin dose requirements [5]. Previous studies in several different ethnic groups, including Thai, have shown that patients with the CYP2C9*2 or CYP2C9*3 allele required lower warfarin doses than those patients with the CYP2C9*1 allele [6–8]. The *VKORC1* gene encodes the VKORC1 enzyme, which is the target enzyme of warfarin action. As reported in several of these ethnic groups, the promoter polymorphism in *VKORC1*-1639G > A, which is, in turn, associated with reduced VKORC1 messenger ribonucleic acid expression, results in the requirement for a lower warfarin dose [7, 9, 10]. The previous studies in several different ethnic groups including Thai populations have reported that patients with single or double variant alleles of *VKORC1*-1639G > A or/and VKORC1 1173C > T required significantly lower warfarin doses compared to patients carrying the wild-type allele [4, 8, 11–13].

A previous study reported that CYP4F2, a vitamin K oxidase enzyme, was involved in the metabolism of vitamin K to hydroxyvitamin K [14]. The carriers of the *CYP4F2* rs2108622 variant allele may have a reduced capacity to metabolize vitamin K and, therefore, require a higher warfarin dose to obtain the same anticoagulant response [15]. The previous studies suggest that *CYP4F2* rs2108622 plays a minor role in warfarin dosage requirement in Caucasians and Asians but not African Americans [3, 16, 17]. In comparison with *CYP2C9* and *VKORC1*, there are a limited number of studies that investigated the effect of *CYP4F2* polymorphism on the warfarin dose requirement in Asians. Recently, the polymorphisms of uridine diphosphate-glucuronosyltransferase 1A1 (*UGT1A1)* rs8175347 and rs887829, which are in linked disequilibrium, were significantly associated with a requirement for higher warfarin doses in Korean and Brazilian patients [18, 19]. The carriers of homozygous *UGT1A1* rs8175347 and rs887829 variant alleles are classified as poor metabolizers [20]. Also, 7-hydroxywarfarin, the major metabolite of warfarin, is conjugated by UGT1A1, which is the phase II drug metabolizing enzyme of warfarin [21]. Therefore, variations in UGT1A1 activity may change the levels of 7-hydroxywarfarin. This previous study has reported that 7-hydroxywarfarin acts as the competitive inhibitor of recombinant CYP2C9 metabolism of *S*-warfarin [22]. Therefore, variations in 7-hydroxywarfarin levels may affect the metabolism of *S*-warfarin. There are few reported data on the association between *UGT1A1* polymorphisms and warfarin doses among multi-ethnic patient populations. Therefore, further study is required to elucidate the impact of *UGT1A1* polymorphisms on warfarin dose requirements in different ethnic groups.

Taken together, non-genetic factors and two main genetic factors, *VKORC1* and *CYP2C9*, only explain approximately 57–58% of the variability in warfarin dosage. Age, weight, height, smoking and interacting medications accounted for less than 20% of the variance [3]. More than 40% of the variance in the warfarin dose requirement remains unaccounted for. Therefore, further studies on additional factors contributing to the unexplained variation are required. Although many studies have investigated the genetic polymorphisms of CYP2C9*3 and *VKORC1* on the warfarin dose requirements, only a few studies simultaneously characterized the effects of all drug metabolizing enzymes in the warfarin metabolic pathway and non-genetic factors in the same cohort study. Thus, the objective of this study was carried out to determine whether non-genetic and genetic factors of relevant drug metabolizing enzymes were involved in warfarin metabolism (CYP2C9*3 and *UGT1A1* rs887829), site of action (*VKORC1*-1639G > A), and vitamin K metabolism (*CYP4F2* rs2108622).

## Materials and methods

### Patients

A total of 250 patients at The Queen Sirikit Heart Center of Northeast Thailand and Srinagarind Hospital of the Faculty of Medicine, Khon Kaen University who had stable doses of warfarin, were retrospectively recruited from the outpatient clinic during 2015–2016. Indications for warfarin in these patients were atrial fibrillation, heart valve replacement, deep vein thrombosis, pulmonary embolism, or chronic thromboembolic pulmonary hypertension. The inclusion criteria of study subjects included: (1) Thai patients were at least 18 years of age; (2) the international normalized ratio (INR) target range was 2.0–3.0; (3) stable weekly doses (SWDs) of warfarin were defined as the constant dose taken at two consecutive clinical visits for at least 1 month within a target INR range of 2–3; (4) patients who had a measured INR that deviated from the target range by ≤0.2 INR units and that did not result in a change in stable warfarin doses were enrolled in the study. Exclusion criteria included: (1) pregnant patients; (2) patients who had laboratory tests showing abnormal liver function tests (AST, ALT ≥3fold the upper limit of normal); (3) patients suffering from thyroid disorders. The study was approved by The Khon Kaen University Ethics Committee for Human Research (HE581068). Written informed consent was obtained from all patients.

### Data collection

The medical records were reviewed. Demographic variables, i.e., gender, age, height, body weight, and body mass index (BMI) and clinical variables, i.e., indications for warfarin, SWDs of warfarin, INR values, concomitant medications, concomitant diseases, alcohol drinking, and smoking status were recorded. Current age was age at the last monitoring point while other covariates such as height, body weight, BMI, comorbidity, and concurrent medications were noted from the stable warfarin dose period.

### Genotyping

Genomic DNAs of study patients were extracted from peripheral blood using QIAamp DNA Blood Mini Kit (QIAGEN, Hilden, Germany). Genotyping of *VKORC1* (−1639G > A; rs9923231; assay ID C_30403261_20), CYP2C9*3 (1075A > C; rs1057910; assay ID: C_27104892_10), *CYP4F2* (1347C > T; rs2108622; assay ID C_16179493_40), and *UGT1A1* (rs887829; assay ID C_2669357_10) was performed by the Taqman allelic discrimination assay using a 7500HT Applied Biosystems thermal cycler (Applied Biosystems, CA, USA).

### Statistical analysis

Deviations from the Hardy–Weinberg equilibrium (HWE) were tested using a chi-square goodness-of-fit analysis. A *p* value of less than 0.01 was assumed to indicate a deviation from HWE. Univariate and multivariate analyses were performed to compare the association of non-genetic factors, e.g., demographic and clinical variables and genetic single nucleotide polymorphisms (SNPs) of interest with stable warfarin doses. Associations between stable warfarin doses and age, weight, height, BMI, and mean INR were evaluated using the Spearman’s correlation. The Mann-Whitney *U* test was used to compare the mean values of warfarin SWDs between two groups. The Kruskal-Wallis *H* test was used to determine associations between the mean values of warfarin SWDs of the three different genotype groups.

Multiple linear regression analysis was used to investigate the factors that independently affected the inter-individual variability of warfarin dose requirements. The variables from univariate analysis with *p* < 0.05 were entered into the multiple linear regression model. In addition, the variables from univariate analyses with *p* > 0.05 (e.g., current smoking status and *CYP4F2* rs2108622 genotype) were entered into the multiple linear regression model. Stepwise selections of variables were entered into the regression method including non-genetic and genetic variables that met the default criteria of *p* < 0.05 for entry and *p* > 0.1 for removal. A 5% two-tailed significance level was used in all tests, and the *p* value of <0.05 was considered significantly different. The relative contributions of non-genetic and genetic factors to the total variability of warfarin dose demands were estimated according to the cumulative square of the correlation coefficient (*R* ^*2*^). The final *R* ^*2*^ of the model indicates the joint contribution of all variables to the variability in therapeutic warfarin dose requirements. The contribution of each factor was calculated as the difference of consecutive cumulative *R* ^*2*^ of each variable. All analyses were performed using SPSS 17.0 software (SPSS Inc., Chicago, IL, USA).

## Results

Demographic and clinical characteristics of 250 patients (129 men and 121 women) are summarized in Supplementary Table 1. The average age of these patients was 60.7 ± 12.8 years (range 22.0–87.0 years). BMI values varied between 14.7 and 43.3 kg/m^2^; the average BMI was 24.2 kg/m^2^. The average of warfarin SWDs was 22.3 ± 9.2 mg. The average INR of all patients was 2.42 ± 0.28. The most common indication for warfarin therapy was atrial fibrillation (67.2%). Over 45% of the study subjects had co-morbid chronic diseases including hypertension, diabetes mellitus, dyslipidemia, and congestive heart failure. From univariate analysis, age, BMI, amiodarone use, antiplatelet drug use, e.g., clopidogrel, prasugrel, and ticagrelor, atrial fibrillation, and deep vein thrombosis/pulmonary embolism/chronic thromboembolic pulmonary hypertension were found to be significantly associated with SWDs of warfarin (*p* < 0.05) (Supplementary Table 1). Gender had no significant effect on SWDs of warfarin (*p* = 0.998) (Supplementary Table 1).

The patients were stratified into four age groups (Table 1). The study results indicated that the patients older than 60 years required significantly lower warfarin SWDs as compared with those in the group of 20–39 years. In contrast, the patients in the group of 40–59 years did not significantly require higher warfarin SWDs than those in the group of 20–39 years (Table 1). Patients’ INR values in the groups of 40–59, 60–79, and 80–99 years were not significantly different from those in the group of 20–39 years (data not shown). Based on the patients’ BMI, patients with class I and II obesity (30–39.9 kg/m^2^) required a significantly 24.9% higher SWDs of warfarin as compared with normal weight patients (Table 1). In contrast, the averages of warfarin SWDs received in the patients who were underweight (< 18.5 kg/m^2^), overweight (25–29.9 kg/m^2^), and class III obesity (morbidly obese) (≥ 40 kg/m^2^) were not significantly different from normal weight patients (Table 1).

**Table 1:** Characteristics of patients receiving warfarin according to the age or BMI categories

Age/BMI category	Age (years)/BMI (kg/m^2^) Mean ± SD (range)	Number (%)	Stable weekly dose (mg) Mean ± SD (range)	*p* value
20–39 years	32.1 ± 4.5 (22.0–38.0)	15 (6.0)	28.0 ± 12.4 (8.0–52.5)	-
40–59 years	51.2 ± 5.5 (41.0–59.0)	88 (35.2)	24.6 ± 9.4 (6.0–55.0)	0.193
60–79 years	67.9 ± 5.5 (60.0–79.0)	133 (53.2)	20.8 ± 8.3 (5.3–43.8)	0.021*
80–99 years	82.5 ± 2.6 (80.0–87.0)	14 (5.6)	15.0 ± 6.1 (10.5–28.0)	0.005*
Underweight	17.3 ± 1.0 (14.7–18.4)	26 (10.4)	18.9 ± 9.2 (6.0–42.0)	0.158
Normal weight	22.4 ± 1.7 (18.5–24.9)	131 (52.4)	21.3 ± 8.7 (5.3–50.0)	-
Overweight	26.9 ± 1.4 (25.0–29.6)	69 (27.6)	23.7 ± 9.3 (6.0–55.0)	0.077
Obese (class I & II)	32.0 ± 1.9 (30.2–37.2)	20 (8.0)	26.6 ± 8.2 (13.5–45.0)	0.008**
Obese (class III)	41.6 ± 1.4 (40.0–43.3)	4 (1.6)	30.5 ± 17.8 (10.5–52.5)	0.266

*P values <0.05 are based on comparisons of the mean warfarin SWDs to the age group of 20–39 years

**P values <0.05 are based on comparisons of the mean warfarin SWDs to normal weight

The non-parametric Spearman-rank correlation analysis of the data revealed that the stable weekly warfarin doses were significantly negatively correlated with age (*r* = −0.332; *p* < 0.001) consistent with the results of the above-mentioned analyses. In contrast, the stable weekly warfarin doses were significantly positively correlated with body weight (*r* = 0.239; *p* < 0.001), BMI (*r* = 0.224; *p* < 0.001). Height and mean INR had no significant correlations with the stable weekly warfarin doses (*r =* 0.111; *P* = 0.81 and *r* = −0.008; *p* = 0.905) (data not shown).

Frequencies of the alleles and genotypes of polymorphisms for *VKORC1*, *CYP2C9*, *CYP4F2*, and *UGT1A1* genes are summarized in Table 2. According to the χ^2^ test for goodness of fit, these four SNPs including *VKORC1* − 1639G > A, CYP2C9*3, *CYP4F2* rs2108622, and *UGT1A1* rs887829 were in HWE at a *p* < 0.01 criterion (data not shown). The comparisons of warfarin SWDs among those patients with different genotypes are shown in Table 2. Patients with AA and GA genotypes of *VKORC1* − 1639G > A required 49.7% and 27.7% significantly lower averages of SWDS of warfarin as compared with those with wild-type GG genotype (*p* < 0.001). The averages of warfarin SWDs were 64.6% and 34.1% lower in CYP2C9 *3/*3 and *1/*3 patients than in *1/*1 patients (*p* = 0.006). The dosage requirement differences, however, were not observed in patients with variant genotypes of *CYP4F2* rs2108622 and *UGT1A1* rs887829 (*p* = 0.172 and *p* = 0.921) (Table 2).

**Table 2:** Differences in stable weekly doses of warfarin among genotypes

Genotype/Allele	Number of patients (%)	Stable weekly dose (mg) Mean ± SD	*p* value
*VKORC1* -1639G > A			
Genotype			
GG	7 (2.8)	38.6 ± 8.0	<0.001
GA	70 (28.0)	27.9 ± 9.0	
AA	173 (69.2)	19.4 ± 7.5	
Allele			
G	84 (16.8)	-	-
A	416 (83.2)	-	
*CYP2C9*			
Genotype			
*1/*1	239 (95.6)	22.6 ± 9.2	0.006
*1/*3	10 (4.0)	14.9 ± 5.3	
*3/*3	1 (0.4)	8.0	
Allele			
*1	488 (97.6)	-	-
*3	12 (2.4)	-	
*CYP4F2* rs2108622			
Genotype			
CC	153 (61.2)	22.2 ± 10.0	0.172
CT	81 (32.4)	21.8 ± 7.4	
TT	16 (6.4)	25.8 ± 8.4	
Allele			
C	387 (77.4)	-	-
T	113 (22.6)	-	
*UGT1A1* rs887829			
Genotype			
CC	192 (76.8)	22.0 ± 9.1	0.921
CT	56 (22.4)	23.0 ± 9.6	
TT	2 (0.8)	22.8 ± 17.3	
Allele			
C	440 (88.0)	-	-
T	60 (12.0)	-	

A multiple linear regression model using the predictors including *VKORC1* − 1639G > A, CYP2C9*3, and *CYP4F2* rs2108622, age, BMI, antiplatelet drug use, amiodarone use, current smoking status explained 51.3% of individual differences in the stable warfarin doses (Table 3). Increasing age, concomitant use of antiplatelet drugs, amiodarone use, variant genotypes of CYP2C9*3, and *VKORC1* − 1639G > A were negatively associated with warfarin dose requirements; while increased BMI, current smoker status, and the TT variant genotype of *CYP4F2* rs2108622 showed positive correlations. The warfarin SWDs were decreased by 9.7 mg and 18.1 mg in the patients with GA and AA genotypes of *VKORC1* − 1639G > A as compared with those with wild-type GG genotype (*p* < 0.001) (Table 3). Similarly, the warfarin SWDs were decreased by 7.9 mg and 25.9 mg in the patients with CYP2C9 *1/*3 and CYP2C9 *3/*3 genotypes as compared with those with wild-type *1/*1 genotype (*p* < 0.001) (Table 3). According to age, warfarin SWDs were decreased by 0.25 mg with each year of aging (*p* < 0.001) (Table 3). For co-medications, warfarin SWDs were decreased by 7.2 mg and 3.4 mg in the patients receiving amiodarone and antiplatelet drugs together with warfarin (*p* < 0.001 and *p* = 0.021) (Table 3), whereas, warfarin SWDs were increased by 0.3 mg for each 1-point increase in BMI. In addition, warfarin SWDs were increased by 4.1 mg in the patients with the TT variant genotype of *CYP4F2* rs2108622 as compared with those with the wild-type CC genotype (*p* = 0.016) (Table 3). Similarly, warfarin SWDs were increased by 3.9 mg in current smokers as compared with non-current smokers (Table 3).

**Table 3:** Multiple linear regression analyses for variables responsible for stable weekly warfarin doses

Entry into model	Predictors	Unstandardized β Coefficients	Cumulative Model *R* ^*2*^ (%)	Contribution (%)	*p* value
	Constant	45.110			< 0.001
1	*VKORC1* (−1639G > A) AA genotype	−18.109	22.5	22.5	< 0.001
2	Age (in years)	−0.250	33.3	10.8	< 0.001
3	*VKORC1* (−1639G > A) GA genotype	−9.745	37.0	3.7	< 0.001
4	CYP2C9*3/*3	−25.921	40.3	3.3	< 0.001
5	Amiodarone use	−7.245	43.3	3.0	< 0.001
6	CYP2C9*1/*3	−7.919	46.0	2.7	< 0.001
7	Body mass index	0.345	48.3	2.3	< 0.001
8	*CYP4F2* rs2108622 TT genotype	4.149	49.5	1.2	0.016
9	Antiplatelet drugs use	−3.359	50.5	1.0	0.021
10	Current smoker	3.968	51.3	0.8	0.039

Variables are defined as follows:

*VKORC1*-1639G > A genotype: Input 1 for GA or AA, 0 for GG; *CYP2C9* genotype: Input 1 for CYP2C9*1/*3 or CYP2C9*3/*3, 0 for CYP2C9*1/*1; Age: Input age in years; Amiodarone use: Input 1 for patient taking amiodarone, otherwise zero; Body mass index: Input BMI in kg/m^2^; *CYP4F2* rs2108622 genotype: Input 1 for TT, otherwise zero; Antiplatelet drugs use: Input 1 for patient taking antiplatelet drugs including clopidoglel, prasugrel, ticagrelor, otherwise zero; Current smoker: Input 1 for current smoker, otherwise zero

## Discussion

Results from the present study suggest that genetic factors, including CYP2C9*3, *CYP4F2* rs2108622, and *VKORC1* − 1639G > A genotypes and non-genetic factors with age, BMI, amiodarone use, antiplatelet drug use, and current smoking explained 51.3% of the variability in warfarin dose requirements. The present study is the first study to simultaneously evaluate the effects of genetic polymorphisms of all drug metabolizing enzymes that are responsible for warfarin metabolism (*CYP2C9* and *UGT1A1*), site of action (*VKORC1*), and vitamin K metabolism (*CYP4F2*) on warfarin dose requirements in Thai patients. Apart from *VKORC1* and *CYP2C9* genes, these results showed that the *CYP4F2* gene also contributed to warfarin dose requirements in Thais at a modest level, whereas *UGT1A1* did not contribute to warfarin dose variability.

In this study, it confirmed the previous findings that *VKORC1* − 1639G > A and CYP2C9*3 were the major genetic determinants of warfarin dose requirements, with CYP4F2 having a smaller effect [3, 23, 24]. This also confirmed the previous studies that at 26.2% of the warfarin dose variance (22.5% from *VKORC1* (−1639G > A) AA genotype and 3.7% from *VKORC1* (−1639G > A) GA genotype) (Table 3) [3, 25], the *VKORC1* − 1639G > A polymorphism accounted for the largest portion. Multiple linear regression was used to examine the associations between multiple variables and stable weekly warfarin doses at one time by adjusting the confounding factors, e.g., age, BMI, antiplatelet drug use, amiodarone use, current smoking, CYP2C9*3, and *CYP4F2* rs2108622, which may affect the *VKORC1*-1639G > A effects on warfarin dose variance.

The contribution of *VKORC1*-1639G > A was greater than that of CYP2C9*3, which was similar to those previous reports [6–8]. The present study showed that the CYP2C9*3 allele could explain about 6.0% of the warfarin dose variance (3.3% from CYP2C9*3/*3 and 2.7% from CYP2C9*1/*3) (Table 3). Similarly, as in the previous studies, the predictive contribution of CYP2C9*3 varied from 1.7–5.4% in Asians [7, 26], which was lower when compared with Caucasian populations [3]. A previous study in a Caucasian population reported that defective *CYP2C9* alleles explained 16.7% of the variability in warfarin dose (12.9% from CYP2C9*3 and 3.8% from CYP2C9*2) [3]. It is possible that no CYP2C9*2 allelic variant and lower frequencies of the CYP2C9*3 were found in Asians as compared with Caucasians [27] which could explain the lower contribution of the CYP2C9*3 allele in this study. For the *CYP4F2* rs2108622 polymorphism, the results revealed that the variant TT genotype was identified as a significant contributor and accounted for a modest part of warfarin dose variability (1.2%) (Table 3). Similarly, the previous studies in Asians and Caucasians reported that the predictive contribution of *CYP4F2* rs2108622 varied from 1.4–4.0% [3, 23, 24]. According to the *UGT1A1* rs887829 polymorphism, the present results showed that *UGT1A1* rs887829 did not significantly contribute to the variability of the stable warfarin doses (Table 2) which was not consistent with the study of Korean patients [18]. In this Thai study population, MAF of *UGT1A1* rs887829 SNP was similar to the Korean population (12% vs. 13.2%) [18]. MAF of *UGT1A1* rs887829 SNP was, however, higher in Caucasians (31%) and African-Americans (43%) [28]. Although the MAF of *UGT1A1* rs887829 in this study population is similar to Koreans, the effects of *UGT1A1* rs887829 on warfarin dose requirements between Thais and Koreans were different. The reason for these different results is still not well understood.

In terms of non-genetic factors, these results were similar to those previously reported in the literature [3, 7, 29], namely age, BMI, current smoking status, and use of co-medications. In comparison to genetic factors, non-genetics factors played a less important role to explain the variability in warfarin dose requirements. Among non-genetic factors investigated, age accounted for the largest portion, 10.8% of the warfarin dose variance (Table 3). Additionally, interacting co-medications including amiodarone and BMI explained 3% and 2.3% of the dose variance (Table 3). Furthermore, antiplatelet drug use including clopidoglel, prasugrel, ticagrelor explained up to 1.0% of the dose variance. These interactions cause a possible increase in the anticoagulation effect of warfarin when combined with antiplatelet effects. Although both amiodarone and antiplatelet drug use, including clopidogrel, prasugrel, ticagrelor, are considered as minor contributors; however, this knowledge may help the clinicians in adjusting the dose of warfarin for patients who receive long-term amiodarone or antiplatelet drug treatment. Current smoking, according to present results, was considered a minor contributor to guide warfarin dose adjustment. It is possible that smoking may potentially cause an interaction with warfarin by increasing warfarin clearance through the induction of CYP1A2, which leads to a reduced warfarin effect [29, 30].

In summary, results in this present study show that genetic and non-genetic factors are important in determining the variability in warfarin response. *VKORC1-1639G > A* and CYP2C9*3 were the strongest genetic predictors of warfarin doses in Thai patients, with *CYP4F2* rs2108622 having a minor effect; with age, amiodarone use, and BMI being important clinical predictors. Therefore, a more accurate prediction of warfarin dose requirements needs to take into account both genetic and non-genetic factors. There were inherent limitations to the retrospective study design. Confounding factors could not be controlled, e.g., diet containing vitamin K. Prospective studies are needed to determine whether prediction of optimal warfarin doses are enhanced, and adverse effects are avoided by using genetic (*VKORC1*, *CYP2C9*, *CYP4F2)* and non-genetic factors, e.g., age, amiodarone use, BMI, antiplatelet use, and current smoking status to help guide the selection of a patient’s initial warfarin and maintenance doses.

## Electronic supplementary material

Supplementary Table 1 (DOCX 16 kb)
