# Supplementary material for: The impact of non-genetic and genetic factors on a stable warfarin dose in Thai patients
Source: Eur J Clin Pharmacol. 2017 May 26;73(8):973–80. doi: 10.1007/s00228-017-2265-8 (PMC5508045; doi:10.1007/s00228-017-2265-8)
Supplement: Supplementary file 1 — (DOCX 16 kb) [file 228_2017_2265_MOESM1_ESM.docx]

**Supplementary** **Table 1** Demographic and clinical characteristics of study patients and stable weekly doses (SWDs) of warfarin

| **Characteristics** | **Number (%) or**  **mean ± SD (range)** | **Stable weekly dose (mg) mean ± SD** | ***p* value** |
| --- | --- | --- | --- |
| **Gender** |  |  |  |
| Male | 129 (51.6) | 22.3 ± 9.6 | 0.998 |
| Female | 121 (48.4) | 22.2 ± 8.9 |  |
| **Age (years)** |  |  |  |
| < 65 years | 144 (57.6) | 24.2 ± 9.3 | <0.001 |
| ≥ 65 years | 106 (42.4) | 19.6 ± 8.5 |  |
| **Body mass index (kg/m^2^)** |  |  |  |
| < 30 kg/m^2^ | 226 (90.4) | 21.8 ± 9.0 | 0.007 |
| ≥ 30 kg/m^2^ | 24 (9.6) | 27.2 ± 9.9 |  |
| **Current smoker** |  |  |  |
| No | 237 (94.8) | 22.1 ± 8.9 | 0.417 |
| Yes | 13 (5.2) | 25.9 ± 13.8 |  |
| **Current alcohol drinker** |  |  |  |
| No | 231 (92.4) | 22.2 ± 9.5 | 0.203 |
| Yes | 19 (7.6) | 23.1 ± 6.1 |  |
| **Indication** |  |  |  |
| **Atrial fibrillation** |  |  |  |
| No | 82 (32.8) | 25.1 ± 10.0 | 0.001 |
| Yes | 168 (67.2) | 20.9 ± 8.5 |  |
| **Valve replacement or valvular heart disease** |  |  |  |
| No | 205 (82.0) | 21.9 ± 9.1 | 0.129 |
| Yes | 45 (18.0) | 24.2 ± 9.9 |  |
| **DVT or PE or CTEPH** |  |  |  |
| No | 236 (94.4) | 21.9 ± 9.1 | 0.006 |
| Yes | 14 (5.6) | 29.0 ± 9.3 |  |
| **Others (e.g. cardiomyopathy,**  **atrial septal defect)** | 23 (9.2) |  |  |
| **Comorbidity** |  |  |  |
| **Hypertension** |  |  |  |
| Absent | 194 (77.6) | 22.3 ± 9.4 | 0.925 |
| Present | 56 (22.4) | 22.2 ± 8.9 |  |
| **Diabetes mellitus** |  |  |  |
| Absent | 215 (86.0) | 22.3 ± 9.3 | 0.956 |
| Present | 35 (14.0) | 22.3 ± 8.9 |  |
| **Dyslipidemia** |  |  |  |
| Absent | 234 (93.6) | 22.2 ± 9.3 | 0.477 |
| Present | 16 (6.4) | 23.7 ± 8.9 |  |
| **Congestive heart failure** |  |  |  |
| Absent | 244 (97.6) | 22.3 ± 9.3 | 0.934 |
| Present | 6 (2.4) | 21.9 ± 9.0 |  |
| **History of stroke** |  |  |  |
| Absent | 231 (92.4) | 22.5 ± 9.4 | 0.099 |
| Present | 19 (7.6) | 19.0 ± 6.9 |  |
| **Concomitant medication** |  |  |  |
| **Amiodarone** |  |  |  |
| No | 234 (93.6) | 22.6 ± 9.3 | 0.033 |
| Yes | 16 (6.4) | 17.2 ± 7.6 |  |
| **Antiplatelet drugs*** |  |  |  |
| No | 226 (90.4) | 22.8 ± 9.2 | 0.003 |
| Yes | 24 (9.6) | 17.6 ± 8.9 |  |
| **Aspirin** |  |  |  |
| No | 214 (85.6) | 22.5 ± 9.3 | 0.372 |
| Yes | 36 (14.4) | 21.1 ± 8.8 |  |
| **Angiotensin converting enzyme inhibitors** |  |  |  |
| No | 212 (84.8) | 22.6 ± 9.3 | 0.147 |
| Yes | 38 (15.2) | 20.7 ± 8.7 |  |
| **Angiotensin II receptor blockers** |  |  |  |
| No | 208 (83.2) | 21.9 ± 9.1 | 0.178 |
| Yes | 42 (16.8) | 24.3 ± 9.6 |  |
| **Beta blockers** |  |  |  |
| No | 114 (45.6) | 23.1 ± 9.4 | 0.198 |
| Yes | 136 (54.4) | 21.6 ± 9.1 |  |
| **Calcium channel blockers** |  |  |  |
| No | 213 (85.2) | 22.3 ± 9.3 | 0.628 |
| Yes | 37 (14.8) | 22.2 ± 9.3 |  |
| **Digoxin** |  |  |  |
| No | 179 (71.6) | 23.0 ± 9.7 | 0.125 |
| Yes | 71 (28.4) | 20.4 ± 7.8 |  |
| **Diuretics** |  |  |  |
| No | 124 (49.6) | 22.1 ± 8.9 | 0.851 |
| Yes | 126 (50.4) | 22.4 ± 9.7 |  |
| **Proton pump inhibitors** |  |  |  |
| No | 213 (85.2) | 22.7 ± 9.4 | 0.065 |
| Yes | 37 (14.8) | 19.7 ± 8.1 |  |
| **Statins** |  |  |  |
| No | 145 (58.0) | 22.9 ± 9.2 | 0.206 |
| Yes | 105 (42.0) | 21.5 ± 9.3 |  |

INR, international normalized ratio; DVT or PE or CTEPH, deep vein thrombosis or pulmonary embolism or chronic thromboembolic pulmonary hypertension

* Antiplatelet drugs include clopidoglel or prasugrel or ticagrelor.
